# Self-Assessed Restorative Care Competence Among Nursing Professionals in Finland: A Cross-Sectional Study

**DOI:** 10.1177/01939459261478193

**Published:** 2026-09-07

**Authors:** Sinikka Lotvonen, Mari Parkkila, Anna-Maria Tuomikoski, Maria Kääriäinen, Vito DiBiasi, Elena Platani, Ilaria Bruno, Heidi Siira

**Affiliations:** 1 Research Unit of Health Sciences and Technology, GeroNursing Centre, University of Oulu, Finland; 2 Northern Ostrobothnia Wellbeing County, Oulu University Hospital, Finland; 3 Research Unit of Health Sciences and Technology, University of Oulu, Finland

**Keywords:** older adults, nursing professionals, restorative care, competence, assessment

## Abstract

**Background:**

Restorative care (RC) has emerged as a promising approach to meet the growing demand for older adults’ social and health services by promoting empowerment, individualization, and functional ability. Self-assessment of RC competence can be a powerful tool for identifying the most important RC learning needs and developing the competence of nursing professionals.

**Objective:**

This study explored the levels and associated factors of nursing professionals’ self-assessed RC competence for older adults in Northern Finland.

**Method:**

Data for this descriptive cross-sectional study were collected between October and November 2023 from nursing professionals (*n* = 438) working in different geriatric care units in a well-being services county in Northern Finland, using the Restorative Care Competence Assessment instrument. Descriptive statistical methods such as means, standard deviations, and frequencies as well as analysis of variance were used to analyze associations between background variations and competence scores.

**Results:**

Total self-assessed RC competence was rated as good (mean approximately 3.4 on a 1-5 scale). Patterns were observed by age and years of experience. Younger and less experienced nursing professionals assessed themselves as more competent in certain competence subscales. Furthermore, differences were observed between types of work unit. Nursing professionals in rehabilitation units reported higher RC competence than those in home care.

**Conclusion:**

Younger nursing professionals with less experience, employed in geriatric units in Northern Finland, assessed themselves as more competent in RC. RC competence should be strengthened through regular continuing education and development of RC models.

## Introduction

Finland has one of the oldest and fastest aging populations in Europe,^1^ with historically 4 generations alive at the same time. It is estimated that the percentage of over 65-year-olds will increase from the current 22% to 26% by 2030 and to 29% by 2060. The country’s national goal is to enable most older adults to live in their own homes for as long as possible.^2^ According to the Finnish Act on Supporting the Functional Capacity of the Older Population and on Social and Health Services for Older Persons,^3^ an older adult is defined as an individual whose physical, cognitive, psychological, or social functional capacity has deteriorated due to diseases or injuries that have begun, increased, or worsened with advanced age, or due to age-related decline.

From the above perspectives, in the future there will be an urgent need to support older adults to live at home safely, prevent or delay admissions to hospital or long-term care, and minimize decline in health and function. At the same time, it is important to promote recovery, improve functional capability, increase independence, promote optimal quality of life, and provide appropriate care when needed.^4^ In the context of aging populations and increasing pressure on health and social care systems, policymakers in high-income countries are seeking cost-effective strategies to support older adults’ independence and reduce reliance on social and healthcare services. To address the growing demand for social and healthcare services, the models of restorative care (RC) and reablement have emerged as promising, function-focused approaches that promote empowerment and individualization.^5,6^

By enhancing older individuals’ capacity to manage daily activities, RC and reablement are commonly grouped under the same umbrella in Finland and internationally, as they share the same underlying care philosophy and are not distinguished from each another, offering potential to delay or prevent the need for more intensive care.^4-7^

RC is a person-centered philosophy of care that focuses on preventing functional decline as well as restoring and maintaining the optimal functional and physical status of older adults. RC can be implemented with all older adults during all care interactions. Traditionally, the focus of the care of older adults’ care settings has been safety and comfort. An RC philosophy adds to that paradigm an emphasis on maximizing and maintaining function.^8,9^ The principles of RC align closely with those in reablement, a widely recognized approach aimed at enhancing and sustaining the functioning of older adults.^5,4,6^

The RC approach includes an initial comprehensive assessment, followed by regular reassessments and the development of an individual goal-oriented RC plan. RC supports individuals to achieve their goals through participation in activities of daily living (ADLs), home modifications, and assistive devices, as well as the involvement of their social network and living environment.^4^ Previous studies have underlined the necessity to change the mentality of older adults’ care from “doing for” to “doing with.”^4,5,10,11^ However, more research on such effective programs is needed. Furthermore, while existing literature has emphasized the importance of multidisciplinary and effective RC competence for older adults,^12-15^ similar research on nursing skills that promote RC is scarce. In clinical practice, there is a gap between theoretical knowledge and its application in older adults’ nursing practice, suggesting that nursing professionals need continuing RC education to master the skills and deal with challenging situations.^11^

Care for older adults is mainly provided by nursing professionals: licensed practical nurses (LPNs) or registered nurses (RNs). LPNs usually work under RNs’ supervision and are primarily responsible for basic patient care, while RNs have greater responsibility for medication management.^16^ Physiotherapists, occupational therapists, social workers, and care assistants also work in the field of older people nursing.^17^ However, among these, LPNs are the largest group of workers in older adults’ social and healthcare services in Finland, with 78% in home care and 66% in round-the-clock care.^18^ Older people nursing is a specialized field of nursing practice based on evidence, experience of nursing professionals, and gerontological competence. It aims to support and promote the physical, mental, social, and spiritual health and well-being of older adults comprehensively.^19^ Competence in nursing care of older adults is a wide-ranging and versatile entity that requires matching complex combinations of knowledge, performance, skills, values, and attitudes.^20^

Self-assessment can be a powerful tool for identifying the most important RC learning needs and developing competencies. When professionals take the time to reflect on their experiences and learning, they can gain a deeper understanding of their strengths and weaknesses, as well as identify areas where they need to improve.^21^ Critical evaluation of care work is an important part of learning and development in an environment where older adults need a lot of care and support.^22^

There is no previous systematic data on nursing staff’s RC competence within the context of care for older adults. In addition, no instrument has previously been used or validated that would allow the collection of broader data across different social and healthcare services for older people. Therefore, this study aims to fill a global knowledge gap regarding nursing professionals’ self-reported RC competence and associated factors. We believe that the findings will help to develop assessment methods for determining the RC competence of nursing professionals and management of RC in geriatric social and healthcare services.

## Aims of the Study

The aim of the study was to explore nursing professionals’ self-assessed competence in RC of older adults and the related factors in Northern Finland. The following research questions were addressed:

1. What are nursing professionals’ self-assessed competence levels in RC of older adults?
2. What are the associations between background variables and nursing professionals’ self-assessed competence in RC of older adults?

## Methods

### Study Design

This was a descriptive cross-sectional study,^23^ and the study reporting follows the Strengthening the Reporting Observational Studies in Epidemiology (STROBE) guide-lines.^24^

### Measures

The Restorative Care Competence Assessment (ReCCA) instrument was developed by researchers at a university in Northern Finland for self-assessment of the RC competence of nursing professionals working in geriatric services in a single well-being service county in Northern Finland. The well-being services county included different geriatric care units, such as home care, nursing homes, assessment and rehabilitation units, home hospitals, and consultation units.

The ReCCA instrument was developed in 3 phases.^25^ In the first phase, the instrument’s conceptual framework was developed, and its items were generated based on literature reviews^5,26,27^ and previous scientific literature on RC,^5,28-31^ as well as the competence needed in nursing professionals working with older adults.^20,32^ Earlier studies have also highlighted the importance of assessing RC performance, particularly nurses’ and carers’ roles, skills, knowledge, and experience.^4,11,12^ In the second phase, the face and content validity of the instrument were evaluated by expert panels and by calculating its Content Validity Index (CVI). The item CVI was computed for each item and for the whole tool (S-CVI/Ave).^33^ In the third phase, the construct validity of the instrument was examined using exploratory factor analysis with principal axis factoring and Varimax rotation with Kaiser normalization.^34^

The ReCCA instrument consists of 5 background questions and 8 subscales. The background questions were the working environment, age, work experience in social and healthcare, work experience in nursing older people, and occupation. In this study, the order of the subscales was changed from the psychometric development phase of the instrument to make it more logical. The new order of subscales were as follows: (1) understanding the impact of values and social factors on RC (4 items), (2) promoting physical functioning (7 items), (3) promoting mental well-being and social capability (4 items), (4) assessing functional capability (4 items), (5) understanding the basic principles of RC (7 items), (6) utilizing resource-oriented and empowering RC (8 items), (7) utilizing safe environment and assistive aids in RC (9 items), and (8) utilizing cooperation and technology in RC ( 5 items). The instrument uses a 5-point Likert scale (1 = poorly, 2 = moderately, 3 = well, 4 = very well, and 5 = excellently) on which the respondents self-assessed their RC competence by indicating the most relevant response among the options.^35^ Response options such as “not applicable” or “not existing” were excluded, as they do not provide meaningful information regarding respondents’ competence. Internal consistency was assessed using Cronbach’s alpha. The subscale values ranged from 0.86 to 0.96, and the overall alpha for the 48-item instrument was 0.98, indicating high reliability (Table 1). An alpha coefficient of >0.70 was considered acceptable.^36^ All item-total correlations exceeded *r* > 0.30, and 92% of inter-item correlations fell within the recommended range (0.30 < *r* < 0.70^25^; see Table 1).

**Table 1.** Subscales and Cronbach’s Alpha of the ReCCA Instrument.

Subscale	*n*	Cronbach’s alpha
ReCCA—instrument total	438	0.98
Understanding the impact of values and social factors on RC (4 items)	438	0.86
Promoting physical functioning (7 items)	438	0.95
Promoting mental well-being and social capability (4 items)	438	0.91
Assessing functional capability (4 items)	438	0.96
Understanding the basic principles of RC (7 items)	438	0.94
Utilizing resource-oriented and empowering RC (8 items)	438	0.94
Utilizing safe environment and assistive aids in RC (9 items)	438	0.95
Utilizing cooperation and technology in RC (5 items)	438	0.91

Abbreviations: RC, restorative care; ReCCA, Restorative Care Competence Assessment.

### Participants

The data were collected from LPNs (*n* = 345) and RNs (*n* = 93) working in different geriatric care units, such as home care, nursing home, assessment and rehabilitation unit, home hospital care, and consultation unit, of a well-being services county in Northern Finland. In this study, the participants are referred to as “nursing professionals.”

### Data Collection

The data were collected electronically using the Webropol 3.0 survey and reporting tool (Helsinki, Finland) between October and November 2023. A contact person from one well-being services county invited all nursing professionals of the selected units to participate in the study by e-mail, providing study information and a link to the Webropol survey. An online survey was chosen because of its cost-effectiveness and efficiency compared to article surveys.^37^ The target group included all LPNs and RNs working in geriatric care services in a well-being services county in Finland, and the online survey was sent to the entire target group (*n* = 2916). The inclusion criteria were as follows: nursing professionals working in home care, nursing homes, or assessment and rehabilitation units. The online survey included information about the study, a question regarding participants’ willingness to take part in the research, background variables, the ReCCA instrument items, and open-ended questions concerning organizational development needs. The participants were also informed about their right to withdraw from the study at any phase without providing a reason. The data collection period lasted for 4 weeks. The total response rate was 15% (*n* = 438), which took 2 reminder e-mails to achieve. The questionnaire was distributed to all healthcare professionals, and participation was based on voluntary self-selection. As the selection process was not random, this may introduce self-selection bias. In this study, the data were collected not only to determine the nursing professionals’ competence but also for the development and psychometric testing of the instrument.^25^

### Data Analysis

The data were analyzed using descriptive statistical methods. The distributions of the sum variables were normal. The items in subscales were rated on a 1 to 5 scale, indicating the label associated with each point. The competence level of nursing professionals in RC was described using mean, standard deviation, percentages, and frequencies. First, the 8 subscales of RC competence of nursing professionals were re-coded into a 3-category variable. This was achieved by combining classes 1 (poor) and 2 (fair) and classes 4 (good) and 5 (excellent). As a result, a new 3-point variable was created (fair = 1-2.499, good = 2.5-3.499, and very good = 3.5-5) and used for the analysis. The chosen cut-off points were based on the theoretical interpretation of the 1 to 5 Likert scale, where values below 2.5 correspond to lower-than-expected competence, values around the midpoint of the scale (2.5-3.49) reflect adequate or good competence, and values ≥3.5 represent high competence. This categorization approach enables consistent interpretation across subscales of varying length (2-11 items) and is commonly applied in multidimensional competence assessments to enhance descriptive interpretability while retaining continuous variables for inferential analyses.^38^

The associations between the mean RC competence scores and the background variables—age, occupation, work experience in social and healthcare, work experience in older people nursing, and working environment—were examined using 1-way analysis of variance.^39^ A *P* < .05 was considered as the threshold of statistical significance. The data were analyzed using SPSS Statistics 27 (IBM Corp, Armonk, NY, United States). The study had no missing data as all questions were set as mandatory for the respondents.

### Ethical Considerations

The study followed the guidelines of the Finnish National Board on Research Integrity.^40^ Research permission to proceed was received from all participating organizations. The Regional Ethics Committee’s permission was not needed as the study did not affect the physical or psychological integrity of the participants. Because the ReCCA instrument was developed by the researchers at the university, its use in this study did not require permission. The participants were sent information about the study and asked about their willingness to participate in the study by e-mail. The participants were also informed about their right to withdraw from the study at any phase without reason. The anonymity of the participants was secured in all stages of the study.^41^ The data protection legislation of the European Union, (EUR-Lex)^42^ such as the General Data Protection Regulation 2016/679, was also followed in the collection and processing of personal data.^10^

## Results

### Characteristics of Participants

Of the 438 participants, 345 (79%) were LPNs and 93 (21%) were RNs. The mean age of the participants was 46 years (range, 18-67 years). Of the total, 200 participants (46%) had worked in older adult nursing for 10 years or more. Examination of the service areas suggested that over 90% of the nursing professionals were employed either in home care (51%, *n* = 222) or nursing homes (39%, *n* = 169) and a smaller proportion worked in assessment and rehabilitation units (7%, *n* = 33) and home hospital care (3%, *n* = 12; Table 2).

**Table 2.** Background Information of Nursing Professionals (*n* = 438).

Background variable	*n*	%
Age (years)		
18-30	51	12
31-50	197	45
51-67	190	43
Working experience in social and healthcare (years)		
<10	165	38
11-20	130	30
21-30	89	20
>30	54	12
Working experience in older adult’s care (years)		
<10	200	46
11-20	123	27
21-30	73	17
>30	42	10
Occupation		
LPN	345	79
RN	93	21
Working environment		
Home care	222	51
Nursing home	169	39
Assessment and rehabilitation unit	33	7
Home hospital	12	3
Consultation unit	2	0

Abbreviations: LPN, licensed practical nurses; RN, registered nurses.

Table refers to nursing professionals including RN and LPN.

### Self-Assessed RC Competence

The total self-assessed RC competence was rated as good (mean = 3.4 on 1-5 scale; standard deviation = 0.72). Almost half (46%) of the participants rated their total RC competence as very good, 43% rated their competence as good, and only 11% rated competence as fair. The participants self-assessed their RC competence as very good in 1 subscale: utilizing resource-oriented and empowering RC (mean = 3.9). In 7 subscales, the nursing professionals rated their self-assessed RC competence as good: utilizing safe environment and assistive aids in RC, assessing functional capability, understanding the impact of values and social factors on RC, promoting mental well-being and social capability, promoting physical functioning, utilizing cooperation and technology in RC and understanding the basic principles of RC (mean = 3.1-3.5). The participants rated their self-assessed competence lowest in understanding the basic principles of RC and in understanding new olde age (mean = 3.1) and as well as in utilizing cooperation and technology in RC (mean = 3.2). Interestingly, 71% of the participants rated their RC competence in utilizing resource-oriented and empowering RC as very good, while the corresponding percentage for promoting physical functioning was only 38%. The most important RC learning needs were in understanding the basic principles of RC and utilizing cooperation and technology in RC (Table 3).

**Table 3.** Nursing Professionals’ Self-Assessed Competence Levels of Restorative Care of Older Adults (*n* = 438).

Subscale	Items	*M* ± SD	Fair, *n* (%)	Good, *n* (%)	Very good, *n* (%)
Utilizing resource-oriented and empowering restorative care	8	3.85 ± 0.67	5 (1)	122 (28)	311 (71)
Utilizing safe environment and assistive aids in restorative care	9	3.47 ± 0.67	27 (6)	197 (45)	214 (49)
Assessing functional capability	4	3.44 ± 0.76	36 (8)	183 (42)	219 (50)
Understanding the impact of values and social factors on restorative care	4	3.38 ± 0.68	23 (5)	196 (45)	219 (50)
Promoting mental well-being and social capability	4	3.37 ± 0.71	33 (8)	194 (44)	211 (49)
Promoting physical functioning	7	3.28 ± 0.70	56 (13)	214 (49)	168 (38)
Utilizing cooperation and technology in restorative care	5	3.16 ± 0.78	98 (22)	202 (47)	138 (31)
Understanding the basic principles of restorative care	7	3.07 ± 0.82	106 (24)	190 (44)	142 (32)
Total restorative care competence	48	3.38 ± 0.72	28 (11)	187 (43)	203 (46)

Competence level (mean): fair = 1 to 2.49, good = 2.5 to 3.49, and very good = 3.5 to 5.

### Associations Between Background Variables and Nursing Professionals’ Self-Evaluated RC Competence

The age of the participants was statistically significantly associated with 4 of the 8 subscales: understanding the impact of values and social factors in RC (*F*_[2, 143]_ = 4.98, *P* = .007, η^2^*p* = 0.02), understanding the basic principles of RC (*F*_[2, 139]_ = 5.70, *P* = .004, η^2^*p* = 0.03), utilizing resource-oriented and empowering RC (*F*_[2, 142]_ = 5.29, *P* = .005, η^2^*p* = 0.02), and utilizing cooperation and technology in RC (*F*_[2, 142]_ = 23.2, *P* = .001, η^2^*p* = 0.01). The results showed that the best self-assessed competence on all 8 subscales was observed in nursing professionals under 30 years of age (Table 4).

**Table 4.** Associations Between Background Variables and Nursing Professionals’ Self-Evaluated Competence in Restorative Care of Older Adults (*n* = 438).

Background variables	*n* (%)	Understanding the impact of values and social factors in restorative care		Promoting physical functioning		Promoting mental well-being and social capability		Assessing functional capability		Understanding the basic principles of restorative care		Utilizing resource-oriented and empowering restorative care		Utilizing safe environment and assistive aids in restorative care		Utilizing cooperation and technology in restorative care	
		Mean (SD)	*P*	Mean (SD)	*P*	Mean (SD)	*P*	Mean (SD)	*P*	Mean (SD)	*P*	Mean (SD)	*P*	Mean (SD)	*P*	Mean (SD)	*P*
Age (years)			.007		.053		.078		.064		.004		.005		.064		.001
18-30	51 (12)	3.66 (0.65)		3.50 (0.69)		3.56 (0.68)		3.66 (0.79)		3.43 (0.85)		4.06 (0.65)		3.67 (0.58)		3.72 (0.73)	
31-50	197 (45)	3.38 (0.66)		3.24 (0.68)		3.40 (0.71)		3.42 (0.77)		3.03 (0.79)		3.89 (0.64)		3.48 (0.68)		3.22 (0.75)	
51-67	190 (43)	3.32 (0.70)		3.25 (0.71)		3.31 (0.72)		3.38 (0.74)		3.02 (0.81)		3.43 (0.68)		3.42 (0.72)		2.95 (0.74)	
Occupation			.579		.196		.903		.482		.203		.372		.358		.972
Licensed practical nurse	345 (79)	3.39 (0.67)		3.20 (0.81)		3.38 (0.75)		3.45 (0.73)		3.09 (0.78)		3.90 (0.68)		3.49 (0.64)		3.16 (0.74)	
Registered nurse	93 (21)	3.35 (0.71)		3.28 (0.70)		3.37 (0.71)		3.39 (0.87)		2.97 (0.93)		3.85 (0.66)		3.42 (0.76)		3.16 (0.90)	
Work experience in social and health care (years)			.077		.647		.654		.952		.803		.543		.781		.001
≤10	165 (38)	3.50 (0.70)		3.31 (0.71)		3.43 (0.70)		3.41 (0.78)		3.08 (0.79)		3.91 (0.68)		3.51 (0.67)		3.38 (0.78)	
11-20	130 (30)	3.31 (0.70)		3.21 (0.68)		3.36 (0.74)		3.46 (0.71)		3.09 (0.80)		3.79 (0.66)		3.43 (0.66)		3.00 (0.71)	
21-30	89 (20)	3.32 (0.63)		3.29 (0.71)		3.35 (0.70)		3.45 (0.77)		2.99 (0.86)		3.83 (0.65)		3.47 (0.65)		3.04 (0.81)	
≥31	54 (12)	3.36 (0.65)		3.32 (0.71)		3.31 (0.75)		3.41 (0.85)		3.09 (0.88)		3.85 (0.67)		3.49 (0.71)		3.07 (0.71)	
Work experience in older adults’ care (years)			.017		.404		.189		.977		.915		.459		.747		.001
≤10	200 (46)	3.50 (0.70)		3.33 (0.71)		3.45 (0.70)		3.44 (0.77)		3.07 (0.80)		3.90 (0.67)		3.51 (0.68)		3.35 (0.79)	
11-20	123 (28)	3.26 (0.65)		3.20 (0.65)		3.32 (0.72)		3.41 (0.73)		3.10 (0.79)		3.78 (0.66)		3.45 (0.64)		2.97 (0.69)	
21-30	73 (16)	3.30 (0.65)		3.24 (0.74)		3.36 (0.70)		3.45 (0.77)		3.01 (0.88)		3.84 (0.66)		3.43 (0.66)		3.04 (0.79)	
≥31	42 (10)	3.36 (0.70)		3.30 (0.73)		3.23 (0.77)		3.44 (0.81)		3.07 (0.87)		3.83 (0.65)		3.47 (0.72)		3.01 (0.75)	
Work area			.042		.008		.254		.035		.016		.257		.367		.001
Home care	190 (46)	3.44 (0.70)		3.23 (0.69)		3.35 (0.72)		3.41 (0.77)		3.03 (0.85)		3.80 (0.65)		3.44 (0.66)		3.28 (0.78)	
Nursing home	192 (44)	3.30 (0.67)		3.30 (0.68)		3.41 (0.70)		3.45 (0.74)		3.10 (0.76)		3.86 (0.69)		3.49 (.67)		2.99 (0.74)	
Rehabilitation unit	33 (7)	3.60 (0.64)		3.57 (0.71)		3.48 (0.71)		3.64 (0.69)		3.33 (0.72)		4.03 (0.63)		3.63 (0.69)		3.32 (0.84)	
Home hospital	14 (3)	3.25 (0.47)		2.87 (0.83)		3.07 (0.72)		2.94 (0.91)		2.53 (1.03)		3.95 (0.59)		3.33 (0.60)		3.37 (0.77)	

Abbreviation: ANOVA, analysis of variance.

The presented results were obtained through 1-way ANOVA-test. Bold values denote statistical significance at the *P* < .05 level. Competence level (mean): fair = 1 to 2.49, good = 2.5 to 3.49, and very good = 3.5 to 5.

Nursing professionals’ work experience in social and healthcare was statistically significantly associated with RC competence in 1 subscale: utilizing cooperation and technology in RC (*F*_[3, 431]_ = 7.43, *P* = .001, η^2^*p* = 0.02). Participants with less than 10 years of work experience in older adult care had better RC competence in 4 subscales than those with more years. Nursing professionals’ working years in older adult care were statistically significantly associated with self-assessed RC competence in 2 subscales: understanding the impact of values and social factors in RC (*F*_[3, 434]_ = 3.44, *P* = .017, η^2^*p* = 0.02) and utilizing cooperation and technology in RC (*F*_[3, 434]_ = 7.94, *P* = .001, η^2^*p* = 0.02). Participants with less than 10 years of work experience in older adult care had better RC competence in 6 subscales than those with more years (Table 4).

The nursing professionals’ work area was statistically significantly associated with RC competence in 5 subscales: understanding the impact of values and social factors in RC (*F*_[3, 432]_ = 2.76, *P* = .042, η^2^*p* = 0.02), promoting physical functioning (*F*_[3, 432]_ = 3.95, *P* = .008, η^2^*p* = 0.03), promoting mental well-being and social capability (*F*_[3, 432]_ = 2.88, *P* = .035, η^2^*p* = 0.02), understanding the basic principles of RC (*F*_[3, 432]_ = 3.48, *P* = .016, η^2^*p* = 0.02), and utilizing cooperation and technology in RC (*F*_[3, 432]_ = 6.72, *P* = .001, η^2^*p* = 0.04). The RC competence of nursing professionals’ working in the rehabilitation ward was better in 7 subscales than those working elsewhere (Table 4).

## Discussion

This study examined nursing professionals’ self-assessed competence across various geriatric units. Overall, the participants’ total self-assessed RC competence was at a good level.

Participants with less than 10 years of work experience in older adult care reported better RC competence than those with more years. Work experience in older adults’ care was significantly associated with RC competence in 2 subscales: utilizing resource oriented and empowering RC and promoting mental well-being and social capability. Moreover, nursing professionals in rehabilitation units reported higher RC competence than home care.

Research has shown that RC training programs have a positive impact on home care staff’s knowledge and skills of and attitude toward RC.^43,44^ The self-assessment instrument used in this study provided valuable insights into nurses’ perceived competence in RC. However, it is important to acknowledge the subjective nature of self-assessment, as previous research has indicated that self-perceptions may not consistently reflect actual competence.^44^ Moreover, the possibility of the Dunning-Kruger Effect—where individuals with lower competence tend to overestimate their abilities, while more competent individuals may underestimate their competence^45^—should be considered as a potential source of bias.^45^ Despite these limitations, self-assessment remains a useful tool in evaluating professional skills and supporting reflective practice.^22^

The ReCCA instrument developed in this study enabled participants to self-assess their RC competence, contributing to their professional awareness of RC competence and its development. One limitation to note is that, due to the novelty of the ReCCA instrument, it has not yet been validated across multiple well-being service counties or in international context.

The participants rated their competence as very good on 1 subscale: utilizing resource-oriented and empowering RC. Utilizing resource-oriented and empowering RC focuses on supporting older adults’ individual resources and is, therefore, one of the key factors in supporting their functional capability.^30^ In this aspect, RC includes targeted efforts based on the older adults’ individual choices and interests, ranging from managing everyday life to being able to participate in activities outside their homes. The importance of person-centered care, including understanding the needs and desires of older adults, is one of the key elements of RC. The person-centered care approach is the person’s right to experience self-determination and equal understanding from nursing professionals.^46^ However, nursing professionals often feel that they do not always have sufficient time for unhurried encounters and getting to know older adults.^47^

Our finding that nursing professionals rated good self-assessed competence in understanding the impact of values and social factors on RC suggests that staff have internalized the value base associated with RC and recognize the importance of interaction, collaboration, and person-centeredness in their work. At the same time, the finding may reflect that the shift from traditional care toward a rehabilitative approach is already well advanced at the level of attitudes and self-perceived competence, even though practical implementation may still be influenced by factors such as resources and organizational conditions.^31^

The nursing professionals self-assessed their competence in promoting older adults’ physical functioning as good. Restrictions on physical functional capability, including ADLs and instrumental ADLs, are the most significant factors that can make independent living at home difficult for older adults, leading to an increased need for home care services and eventual transition to a nursing home.^48^ Nursing professionals’ role in promoting functional capability and preventing further disability is crucial. Research has shown that RC training programs for home care staff are effective in improving the physical functioning of older adults.^49^ RC care focuses on self-management and independent living in the home environment, and it includes mobilization and self-care training (eg, independent washing, toileting, and eating).^50^ Likewise, nursing professionals’ competence in promoting mental well-being and social capability of older adults was rated as good. Accordingly, psychosocial empowerment and activation, as well as engagement of older adults’ social networks to support their participation in meaningful activities, are key components of RC.^4,51^

In our study, the nursing professionals rated their competence in assessing older adults’ functional capability at a good level. RC includes comprehensive assessments, goal-oriented care, and regular follow-up assessments and care plan reviews.^4^ Nursing professionals should be trained to effectively assess RC needs and to use assessment data for RC goal setting as well as the planning and delivery of RC.^49,50^ To achieve this, it requires investment in RC education and training programs. Nursing professionals should be given access to up-to-date RC knowledge to enable them to make evidence-based decisions that focus on older adults’ individual conditions and needs.^32^

Our finding that nursing professionals reported good understanding of the basic principles of RC aligns with previous literature emphasizing that such understanding reflects competence in person-centered RC, as well as in identifying goals and RC planning, In practice, this means that professionals need clear RC models to translate their knowledge into consistent goal setting, documentation, and follow-up, which are essential for improving clients RC an individualized RC plan on which RC is based.^29^

Likewise, nursing professionals’ competence in utilizing safe environment and assistive aids in RC was rated good. Considering that disability represents a mismatch between the living environment and older adults’ functional capability, nursing professionals can help older adults restore their function by reducing environmental demands.^46^ Simple home modifications, such as installing bathroom safety equipment and shower seats, can reduce older adults’ barriers to independence.^51^ Furthermore, identifying environmental barriers, such as the absence of non-slip mats and too-high microwave placement, and improving safety using assistive aids is essential for preventing functional limitations and disabilities.^52^

Nursing professionals’ RC competence in utilizing cooperation and technology was at a good level. This includes understanding the importance of multidisciplinary cooperation as well as counseling older adults and their loved ones to use various technological solutions and equipment in daily life.^4^ Similarly, it refers to nursing professionals’ competence in utilizing modern technological solutions to support RC.

The results indicated that participants’ age was significantly associated with 4 of the 8 subscales. This finding is consistent with earlier research that showed a rapid increase in nursing professionals’ competence level during the first 10 years of their clinical experience.^53^ Nursing professionals with less than 10 years of experience in social and healthcare reported statistically significantly higher RC competence in 1 subscale—utilizing cooperation and technology in RC—compared with those with more experience. There was also a significant association between nursing professionals’ working years in older adult care and self-assessed competence in understanding the impact of values and social factors on RC and utilizing cooperation and technology in RC. Similar to these findings, a recent study reported highest competence levels among nursing professionals with 5 to 10 years of experience in older adult care.^54^ The first 10 years of clinical practice provide extensive learning opportunities through new knowledge, skills, and attitudes gained both individually and from colleagues.^53^ Newly graduated nursing professionals may, in some cases, overestimate their RC competence, although it usually develops over time with professional experience. Reportedly, nursing professionals with more than 10 years of work experience are at risk of stopping at the current level of competence.^53^

There was a significant association between nursing professionals’ working area and self-assessed competence in understanding the impact of values and social factors in RC, promoting physical functioning, assessing functional capability, understanding the basic principles of RC, and utilizing cooperation and technology in RC. The self-assessed RC competence of nursing professionals working in the rehabilitation ward was better than those working in other units in 7 subscales. This might be explained by the multidisciplinary collaboration in the rehabilitation wards, which presents opportunities for various perspectives among colleagues who may have different insights due to their professional backgrounds, training, and experiences. Such dynamic multiprofessional interaction has been shown to enhance a higher standard of professional competencies.^55^ In the rehabilitation unit, the care for older adults is focused on assessment, goal setting, and implementation of rehabilitation and RC. In home hospital care, the goal of the care is less restorative, primarily focusing on acute geriatric conditions and their management, in addition to nursing professionals working mostly alone.

Continuing educational investments, such as giving nursing professionals access to evidence-based continuing education online courses in RC, are necessary to strengthen their knowledge and development in RC. However, because a major part of their learning occurs during everyday practices, the establishment of RC models that support learning from experience would be beneficial. Nurse leaders play an important role in creating effective learning environments, enabling continuing education, and ensuring excellent RC.

### Strengths and Limitations

One of the strengths of this study is that it provides insights into the RC competence of nursing professionals working in different geriatric care units of a well-being services county in Northern Finland. Second, the new ReCCA instrument was developed and used for the first time in this study to collect data on nursing professionals’ RC competence in older adult care. We demonstrated that the ReCCA instrument can be used in geriatric care units for self-evaluation and competence development in RC.

The limitation of this study is that despite great efforts put into encouraging the nursing professionals to participate in the study, including sending reminders, the response rate was only 15%. This limitation makes it difficult to draw firm conclusions and comparisons from the results. Reportedly, online questionnaires generally result in lower response rates.^56^ However, the sample size was large enough for the analysis performed.^57^ The results of the study should be seen critically because it is possible that the instrument was mainly answered by nursing professionals who were most motivated and competent in RC of older adults. As the data collection system required responses to all items, no missing data were generated. This methodological choice may, however, have led some participants to provide less reflective answers to items they perceived as less relevant or more difficult to interpret.

The findings of this study highlight the need to strengthen the RC competence of nursing professionals across all older adults’ care settings. Nurse leaders play an essential role in creating supportive learning environments, fostering interdisciplinary collaboration, and enabling access to continuing education. Training programs should emphasize systematic RC education, including assessment, goal-oriented practice, environmental modification, and cooperation with relatives. At the service-system level, reinforcing RC-oriented models and monitoring staff competence—for example, using the ReCCA instrument—can help ensure consistent quality of care. Future research should examine RC competence in diverse care environments internationally and explore the relationship between self-assessed and externally assessed competence. Although the ReCCA instrument has undergone psychometric testing,^25^ further validation is still warranted. Specifically, we now highlight that additional studies are needed to confirm the instrument’s psychometric properties across different populations and contexts.

## Conclusion

Nursing professionals’ self-assessed RC competence was generally good but varied across care units, with assessment and rehabilitation units reporting the highest levels. Competence in utilizing cooperation and technology in RC and understanding the basic principles of RC, utilizing cooperation and technology in RC, requires further development. The highest competence levels were reported by younger professionals and those with shorter experience in social and healthcare and older adults’ care. Newly graduated nurses may overestimate their RC competence, although it typically develops with professional experience. These findings should be interpreted cautiously due to the low response rate and potential self-selection bias. The reliance on self-assessed data, the forced-response format, the single well-being services county context, and the primarily univariate analytical approach limit the generalizability of the results. Strengthening the integration of RC into older adults’ care and supporting competence development through continuing education are essential, with nurse leaders playing a key role in enabling regular training and effective learning environments.

## Data Availability

The datasets generated and/or analyzed during the current study are not publicly available but are available from the corresponding author on reasonable request.
